# Endourological treatment of upper tract urinary disease in children

**DOI:** 10.3389/fruro.2023.1150795

**Published:** 2023-04-18

**Authors:** Darren Ha, Kelly T. Harris, Kyle O. Rove

**Affiliations:** ^1^Pediatric Urology Research Enterprise, Department of Pediatric Urology, Children’s Hospital Colorado, Aurora, CO, United States; ^2^Division of Urology, Department of Surgery, University of Colorado Denver Anschutz Medical Campus, Aurora, CO, United States; ^3^Department of Pediatric Urology, Children’s Hospital Colorado, Aurora, CO, United States

**Keywords:** endourolgy, pediatrics, megaureter, UPJ obstruction, nephrolithiasis

## Abstract

With advances in therapeutic interventions, endourology has become standard of care for the treatment of numerous diseases in the field of pediatric urology. However, there remains a lack of agreement and evidence on the optimal approaches and associated complications of endourological treatment of upper urinary tract conditions in children, namely ureteropelvic junction (UPJ) obstruction, primary obstructive megaureter, and nephrolithiasis. While pyeloplasty remains the first-line surgical treatment for pediatric UPJ obstruction, endoscopic retrograde balloon dilatation (ERBD) and endopyelotomy continue to gain traction as less invasive means of treating obstruction, particularly for failed repairs. Studies report success rates ranging from 76–100% although re-stenosis or need for revision surgery is not uncommon. Endourological options for the surgical management of primary obstructive megaureter include ERBD or endoureterotomy, rather than the open option of ureteroneocystotomy with or without tapering. Both have shown long-term success rates ranging from 70–90%, however, there is emerging evidence that these therapies may be associated with a risk of postoperative vesicoureteral reflux. Meanwhile, for stone disease, shock wave lithotripsy (SWL), flexible ureteroscopy (URS), and percutaneous nephrolithotomy (PCNL) are mainstays in the pediatric urologist’s armamentarium. Studies have shown that URS and PCNL have comparable stone-free rates, although PCNL can be associated with increased morbidity. Advancements in technology have led to the use of smaller access sheaths without compromising stone-free rates or increasing long-term complications. The use of mini-PCNL in the adult population holds great potential for use in our pediatric patients. The rise of endourology expertise and improved technology makes it an attractive option that could even be considered as a first-line option for the treatment of various urinary tract conditions. Nevertheless, there is a paucity of evidence on outcomes and complications following its use for treatment of upper urinary tract diseases in children. This review aims to summarize and present results of endourological treatments for pediatric UPJ obstruction, primary obstructive megaureter, and nephrolithiasis, as well as highlight advancements in the field of endourology that may increase its utilization in pediatric urology in the future.

## 1 Introduction

Endourology involves the use of specialized instruments and expertise to access both the upper and lower urinary tracts with minimal or no incisions (1). Initially pioneered for use in adult populations, the adoption of endourologic techniques in pediatric urology cautiously trailed behind due to discrepancy in size of instruments and limited scientific studies, among other factors (2). The use of endourology principles in pediatrics has evolved into a variety of tools and techniques to manage urinary tract conditions in children. Surgical management of upper tract diseases, such as ureteropelvic junction (UPJ) obstruction and primary obstructive megaureter (POM), had historically been performed open; while this remains a reasonable option today, particularly for younger children due to body size, the promise of a minimally-invasive option has spurred innovations in endourology that have led to approaches with comparable success rates (3–5). Similarly, the increasing prevalence of pediatric stone disease over the years has driven advancements to adequately treat stones and limit recurrence in children (6). Despite encouraging studies on the safety and efficacy of endourology in the management of upper urinary tract diseases in children, there remains a lack of robust long-term data. This calls for pediatric urologists to have a clear grasp of the current data while keeping an eye toward future efforts to improve the field.

## 2 Ureteropelvic junction obstruction

UPJ obstruction is the most common cause of antenatal hydronephrosis, with reported incidence of up to 1 in 2,000 (7). The ubiquity of prenatal ultrasound has led to hydronephrosis being the most common urologic abnormality detected and this is often the first indication of UPJ obstruction (3). While conservative management with observation has been recommended if MAG3 renogram findings indicate > 40% differential renal function, surgery is the only treatment with curative intent (8). The dismembered open pyeloplasty has been the gold standard surgical treatment for UPJ obstruction since it was first described by Anderson and Hynes in 1949 (9). However, there has been a steady increase in utilization of minimally invasive surgery (i.e., laparoscopic and robotic-assisted pyeloplasty), particularly in the pediatric population, within the last few decades (10, 11). Notwithstanding, endourological techniques, such as endoscopic retrograde balloon dilatation (ERBD) and endopyelotomy, remain as options for a less invasive means of treating obstruction for failed repairs (3).

In 1994, Bolton et al. reported a case study on the use of retrograde endopyelotomy for the treatment of UPJ obstruction in two boys. The main postoperative symptoms were bladder spasms and dysuria, but otherwise healing was uneventful. Intravenous pyelogram (IVP) one year after surgery demonstrated normal urinary drainage without evidence of hydronephrosis in both patients (12). Sugita and colleagues followed with a study evaluating the effectiveness of ERBD for the treatment of UPJ obstruction in 17 children. Eight patients (47%) were successfully treated; six had recurrent or persistent stenosis and the catheter could not be successfully passed in the remaining three patients (13). To circumvent difficulties with retrograde catheter manipulation, Tállai et al. utilized percutaneous antegrade endopyelotomy in 37 children with an 89% success rate after one year as assessed with IVPs. Notably, one patient ultimately underwent pyeloplasty for procedure failure and another required nephrectomy due to bleeding complications (14). In particular, when performing endopyelotomy, the surgeon must be aware of the anatomic relationship between the ureter and the posterior renal vessels or an anterior crossing vessel to avoid bleeding complications, which are discussed further below (15).

In a recent study, Ordóñez et al. evaluated the long-term effectiveness and complications of ERBD for the treatment of primary UPJ obstruction in 112 children with a mean follow-up of 66 months. They reported a 76.8% success rate after one dilatation. Seven required re-dilatation, resulting in a success rate of 86.6% after two dilatations. Ultimately, two patients experienced total loss of renal function and seven required open pyeloplasty. The authors concluded that ERBD is a feasible option for patients, especially given the short procedure time (< 30 minutes), although the main disadvantage of this approach is the likelihood of needing repeat interventions (16). While the success rate of ERBD as a first-line intervention for UPJ obstruction appears comparable to pyeloplasty, these experiences are small, dated, and have relatively short followup. Larger studies with longer-term follow up of associated complications are warranted.

Despite their acceptance as a treatment option, there are limited studies on the role of endopyelotomy or ERBD as salvage treatments following failed pyeloplasty. A 2007 study of 32 children with recurrent UPJ obstruction demonstrated that endopyelotomy had much lower success rates compared to redo pyeloplasty (39% vs 100%, p=0.002). Patient age ≤ 4 years and narrowed ureteral segment > 10 mm were associated with poor outcomes in those treated with endopyelotomy (17). **Table 1** summarizes outcomes of endoscopic management of UPJ obstruction, including after failed pyeloplasty. The minimally invasive nature of endourologic re-treatment following suboptimal results from pyeloplasty may be appealing, but additional work is necessary to fully characterize outcomes, which will ultimately drive informed decision making for patients and their caregivers.

**Table 1:** Summary of outcomes of endoscopic management of ureteropelvic junction (UPJ) obstruction.

Reference	Number of patients	Patient age range	Indication(s)	Treatment(s)	Mean length of follow-up	Success rate	Complications
**Bolton et al. (1994) (** 12)	2	4-6 years	UPJ obstruction	Retrograde endopyelotomy	11.5 months	100%	Urinary frequency, bladder spasms, dysuria
**Sugita et al. (1996) (** 13)	16	29 months (mean)	UPJ obstruction	ERBD	25 months	47.1%	Inability to pass catheter through UPJ, persistent and/or recurrent stenosis
**Tállai et al. (2004) (** 14)	37	4.5-17 years	UPJ obstruction	Antegrade endopyelotomy	12 months	89.0%	Renal drain slid back into renal pelvis, bleeding
**Braga et al. (2007) (** 17)	18	2-14 years	Failed pyeloplasty	Retrograde endopyelotomy	47 months	39.0%	Urinary ascites
**Kim et al. (2012) (** 18)	37	2.7-17 years	UPJ obstruction	Antegrade and retrograde endopyelotomy	34 months	65.0%	Distal ureteral stricture, stent required repositioning
**Ceyhan et al. (2020) (** 19)	21	45.9 ± 46.4 months (mean ± SD)	Failed pyeloplasty	ERBD, retrograde endopyelotomy	46.9 months	29.4% ERBD, 38.8% retrograde endopyelotomy	Urinary tract infection
**Ordóñez et al. (2022) (** 16)	112	13.1 ± 21.3 months (mean ± SD)	UPJ obstruction	ERBD	66.7 months	76.8% after one dilatation, 86.6% after two dilatations	Urinary tract infection, pain, vomiting, hematuria, pyelonephritis, total loss of renal function

ERBD, endoscopic retrograde balloon dilatation.

As with any intervention, endourologic management of UPJ obstruction is not without risks, particularly bleeding due to crossing vessels at the UPJ. Crossing vessels have been cited as a source of extrinsic compression leading to obstruction (20). An early study found that crossing vessels were implicated in two out of three cases of recurrent obstruction (21). More recent studies have identified the presence of crossing vessels in 38–58% of children in their cohorts (18, 20). Additionally, a review found that previously undetected crossing vessels accounted for 31% of failed primary endopyelotomies, which represents a significant proportion of children who may need a repeat procedure. Despite this known risk, very few studies screen for crossing vessels prior to endourologic intervention (22). Thus, pyeloplasty, whether laparoscopic or robotic, offers the advantage of being able to detect crossing vessels at the UPJ and mitigate subsequent bleeding complications with a vascular hitch (20).

## 3 Primary obstructive megaureter

POM is a functional obstruction in a segment of the ureter with subsequent proximal dilatation that may extend up to the renal pelvis (23). Surgical management is indicated in cases of persistent obstructive patterns on MAG3 renogram with at least one of the following: < 40% renal function, worsening renal pelvic dilatation, febrile UTI despite prophylactic antibiotics, and/or presence of renal calculi (4, 24). The initial treatment for POM in children was first described in 1969 and consisted of ureteral reimplantation with tapering (25). The following year, watchful waiting was recommended (26). It was not until 1998 that the first report of endoscopic retrograde balloon dilatation (ERBD) emerged as a novel treatment for obstructive megaureter. This initial study included 11 children, of which six improved after a single dilatation, while the remaining five required two dilatations (27). Since then, there has been limited work scrutinizing treatments for POM in children.

In 2007, Angerri and colleagues evaluated the effectiveness of ERBD as a treatment for POM in seven children, with a mean follow-up of 31 months. Five out of seven patients had reductions in the obstructive pattern on MAG3 renogram after one dilatation, while one patient required a second dilatation to see an improvement (28). That same year, endoureterotomy was described as a new approach to the treatment of POM in children. 47 children with a history of failed conservative management were treated with endoureterotomy and followed for an average of 39 months. The authors reported a success rate of 90%, defined as resolution or decrease in hydroureteronephrosis and improvement or stability in renal function as assessed *via* renal scan. In 71% of patients, there was complete resolution of hydroureteronephrosis. No leakage, obstruction, or reflux was observed in any of the patients (29). As endoscopic technology improved, laser incision and cutting balloons became adjuncts to ERBD for the treatment of longer obstructions or persistent stenosis, with success rates of 70–83% without complications (30–32).

While endoscopic techniques have gained popularity, longer term studies on efficacy and complications remained limited. A 2015 study by Bujons and colleagues evaluated long-term outcomes of ERBD in 19 patients with a mean follow-up of 69 months. They found a 90% initial success rate after one dilatation, with four of nineteen (21%) requiring repeat dilatation. At the end of the study, all patients had significant improvement in hydroureteronephrosis (p<0.001). One patient developed a febrile UTI postoperatively and was discovered to have vesicoureteral reflux (VUR) on voiding cystourethrogram (VCUG) (24). This was the earliest report of postoperative VUR as a potential complication of ERBD. Subsequently, a group of investigators aimed to identify the rate and risk factors associated with VUR after ERBD in 20 children. All patients had VCUGs performed 12 months after surgery, which identified six ureters (27.2%) with VUR: four required surgical repair while two cases resolved spontaneously. Reassuringly, re-dilatation did not seem to affect incidence of VUR. Given that postoperative VCUGs are not routinely performed in children unless indicated, the authors concluded that postoperative VUR is not clinically significant unless accompanied by febrile UTIs, which none of the patients in the study developed (33, 34).

There have been numerous recommended treatments for primary obstructive megaureter in children throughout the decades, beginning with ureteral reimplantation and progressing to recent enthusiasm for endoscopic techniques including ERBD and endoureterotomy. However, guidelines do not offer a clear consensus for recommended therapy. While short-to medium-term success rates with endoscopic modalities have ranged from 76–100%, there is a paucity of long-term studies to reinforce the durability of this repair (4, 29, 32). A systematic review suggested that endoscopic techniques were more effective in children ≥ 12 months of age; in younger patients, this approach may serve as a temporizing intervention prior to ureteroneocystotomy (4). What is apparent in the current literature is that the need for repeat dilatation is not uncommon in order to achieve durable results (4, 24, 27, 28, 33). Thus, appropriate preoperative counseling is necessary for patients and caregivers who are considering current endourological options. While there have been few reported adverse events, there is emerging evidence that postoperative VUR may be a potential complication (24, 33). However, there is insufficient current evidence to conclusively recommend routine postoperative VCUG as a screening modality.

## 4 Pediatric nephrolithiasis

There has been a rising incidence of pediatric nephrolithiasis with reports ranging from a 4–10% increase in incidence annually (35, 36). Proposed drivers of this trend include increased BMI, high sodium diet, low calcium intake, and increased use of computed tomography leading to greater detection. Metabolic, anatomic, and genetic abnormalities also contribute to initial presentation and recurrence of stone disease. While most small stones pass spontaneously in children, α-blockers are a reasonable option for medical expulsion that has been shown to significantly increase the odds of stone passage (6, 37). For those patients who require surgical intervention, endoscopic approaches such as shock wave lithotripsy (SWL), flexible ureteroscopy (URS), and percutaneous nephrolithotomy (PCNL) have largely replaced open procedures (6, 38).

### 4.1 Shockwave lithotripsy

SWL may be offered as first-line treatment for stones of all sizes as it is the least invasive option and there is a shorter skin-to-stone distance in children (38). Reported stone-free rates range from 43–92% (39–42). The American Urological Association (AUA) indicates that SWL is an acceptable option for stones of any size or location in children, including those with a total renal stone burden > 20 mm (43). However, a 2015 meta-analysis showed stone-free rates with SWL were significantly higher with stones < 10 mm and those located in the proximal ureter (44). Regardless, this approach has been associated with a relatively higher rate of retained stone fragments and need for re-treatment; some studies have shown re-treatment rates ranging from 30% to as high as 55% (39, 41, 42). Additionally, steinstrasse and abdominal colic were major complications among 14 published studies, with an overall incidence of 6% and 6.29%, respectively (44, 45).

### 4.2 Flexible ureteroscopy

For ureteral stones or renal stone burden < 20mm, URS is a reasonable choice, with similar efficacy and safety when compared to use in adults (46). The AUA recommends URS for ureteral stones that have failed observation/medical management or for a total renal stone burden ≤ 20 mm, similar to endorsements from others (43, 47, 48). When surveyed, pediatric urologists cited a ≥ 6 mm distal ureteral stone as an indication for URS. However, when presented with a modest decrease in stone size (5 mm), there was no clear consensus on an optimal treatment plan, with hydration alone, hydration with medical expulsion therapy, and URS all equally represented, demonstrating the nuances that exist when choosing an appropriate treatment modality (49). While URS has success rates of 61–98%, larger stone size and lower calyx stone have been shown to be negative predictors of success (42, 46, 50–57). Reassuringly, this approach has been associated with minimal intraoperative complications (50, 52). Meanwhile, reported rates of postoperative complications have ranged from 2.6–17.5%, with UTI, hematuria, and pain being the most common adverse events (42, 50, 54, 56, 58). Additionally, when compared to SWL, URS has been associated with a greater likelihood of 30- and 90-day emergency department visits and 30-day readmissions (39).

### 4.3 Rigid and semi-rigid ureteroscopy

While flexible ureteroscopy has gained popularity and tends to be preferred in the pediatric population, rigid and semi-rigid URS are still utilized, particularly with distal ureteral calculi (58). Early studies of rigid URS in children demonstrated > 90% stone-free rates although ureteral dilatation was required in approximately one third of cases (59, 60). Another study found that the rigid ureteroscope could be easily passed in a majority of children in their cohort, but 17% required conversion to a flexible ureteroscope (61). Semi-rigid URS has also been shown to safe and effective, with complication and stone-free rates comparable to flexible URS (62–64). However, the success of these approaches have mainly been demonstrated for distal ureteral stones, as indicated by greater stone-free rates and fewer complications compared to their use for proximal stones (63). The use of rigid URS is limited by its larger caliber scope and potential risk of ureteral trauma (61). Similarly, semi-rigid URS has been discouraged as a first-line option for proximal ureteral stones (63). Thus, flexible URS grants the greatest range of use, including in proximal ureteral and lower calyx stones (64).

### 4.4 Percutaneous nephrolithotomy

For renal stone burdens > 20 mm, PCNL is an alternative to SWL in children (43, 65). Stone-free rates for PCNL are among the highest of all endourologic stone extraction techniques, ranging from 73.6–95.5% (55, 66–68). One study found that a greater number of stones and larger stone size were associated with decreased stone-free rates (68). Bleeding requiring transfusion is a known complication of PCNL occurring in 2.2–17% of patients, although there have not been any consistent predictors of the need for transfusion, with some studies citing no associations while others noting that increasing stone size was associated with greater risk (67–69). Adapting this technique from the adult population has meant dilating access tracts to 24–30 French. However, there have been concerns about the use of adult-sized instruments for pediatric patients because of the potential risk of complications with the large size of these instruments relative to children’s body and kidney sizes (45). Several studies have found an association between dilation > 22 French and an increased risk of bleeding (66, 69).

Efforts to decrease the size of percutaneous access have led to more size-congruence among pediatric patients, with the mini-PCNL offering 11–20 French caliber access (66). A 2017 systematic review by Jones et al. found stone-free rates ranging from 85–100% with mini-PCNL, which have been corroborated with more recent publications. These promising stone-free rates were accompanied by modest complication rates ranging from 13-17% (42, 57, 70). More recent advances have led to the utilization of 4.8 French access with the micro-PCNL. First used in adults in 2011 and adapted for the pediatric population in 2015, this modality has shown similar success rates to traditional PCNL, with stone-free rates ranging from 80–100% (45, 54, 56, 70). Complication rates are not significantly different from other techniques, ranging from 6-13% (54, 56, 70).

### 4.5 Additional considerations of treatment

URS and PCNL are both reasonable options for ureteral and renal stones and have been shown to be equally effective, stone size and location being equal (42, 54, 56, 57). However, when deciding between these two options, physicians and families should consider additional factors in their decision making process, including bleeding risk, hospital length of stay (LOS), and radiation exposure. Blood loss, irrespective of the need for transfusion, is a not uncommon sequela of PCNL that may significantly influence the approach to stone extraction. A randomized control trial found that blood loss was greater in PCNL compared to URS (55). Various other studies have found that the use of multiple tracts in PCNL has been associated with increased blood loss (66, 67). This, coupled with the fact that there are reports that up to 40% of children require multiple tracts, means that a significant proportion of patients may be at risk of bleeding (69). Furthermore, compared to PCNL, URS has been associated with decreased radiation exposure and hospital length of stay, with Halinski et al. reporting LOS of 3 days for URS versus 4.5 days in PCNL (p<0.001) (42, 54, 56, 57). Thus, while stone-free rate is an important metric for pediatric nephrolithiasis treatments, it should not be evaluated in isolation, but rather in the context of inherent risks of the approach, patient comorbidities, and physician experience (71). **Table 2** provides a summary of outcomes of endourologic approaches to pediatric stones.

**Table 2:** Summary of outcomes of various endourologic treatments for pediatric stone disease.

Reference	Number of patients	Patient age range	Stone size and/or location	Treatment(s)	Stone-free rates	Complication rates
**Shokeir et al. (2006) (** 41)	166	0.6-14 years	1 to 2 cm	SWL, PCNL	45.0% SWL; 86.6% PCNL	1.1% SWL; 4.9% PCNL
**Saad et al. (2015) (** 55)	38	1.42-16.0 years	>2 cm, renal	URS, PCNL	71.4% URS; 95.5% PCNL	9.5% URS; 40.9% PCNL
**Baş et al. (2016) (** 56)	45	8.39 ± 4.72 years (mean ± SD)	1 to 2 cm	Micro-PCNL	80.0%	13.3%
**Çitamak et al. (2016) (** 68)	294	8.51 ± 4.91 years (mean ± SD)	0.4 to 5 cm	PCNL	73.1%%	24.1%
**Farouk et al. (2018) (** 72)	108	2-12 years	1 to 2 cm, pelvic or calyces	SWL, mini-PCNL	55.6% SWL; 88.9% mini-PCNL	14.8% SWL; 22.2% mini-PCNL
**Senocak et al. (2018) (** 67)	97	5 years (3-9); median (IQR)	1 to 2 cm	PCNL	80.9%	5.71%
**Anbarasan et al. (2019) (** 52)	21	2-16 years	0.5 to 3.5 cm	URS	95.0%	0%
**Grabsky et al. (2021) (** 73)	124	10.0 ± 6.0 years (mean ± SD)	<2 cm	SWL	88.0%	0%
**Halinski et al. (2021) (** 54)	53	1.5-18 years	1 to 2 cm	URS, micro-PCNL	84.2% URS; 86.7% micro-PCNL	2.6% URS; 6.0% micro-PCNL
**Ozkent et al. (2021) (** 46)	55	7.2 ± 5.3 years (mean ± SD)	1 to 2 cm	URS	81.8%	13.8%
**Juliebø-Jones et al. (2022) (** 50)	23	1-17 years	0.3 to 4 cm	URS	61.0%	17.5%
**Mahmoud et al. (2022) (** 57)	90	3-14 years	1 to 3 cm	URS, mini-PCNL	88.9% URS; 95.6% mini-PCNL	6.7% URS; 15.6% mini-PCNL

SWL, shock wave lithotripsy; URS, flexible ureteroscopy; PCNL, percutaneous nephrolithomy.

As endourological techniques and technology have continued to evolve, minimally invasive options are often the preferred treatment modality for pediatric stone disease. Nonetheless, there is no clear consensus on ideal endourologic technique, particularly for cases where stone size and/or location may be amenable to multiple approaches. **Table 3** briefly summarizes and spotlights the nuances in recommendations for treatments from the American, Canadian, and European Urological Associations. Regardless of which option is pursued, each has its risks and benefits which should be thoroughly discussed with patients and families to engage them in joint decision making. Pediatric nephrolithiasis can be a difficult condition to manage due to the possibility of recurrence, thus a multidisciplinary approach involving pediatric urologists and pediatric nephrologists may yield the most benefit for children (49). Additionally, preventing recurrence involves modifying risk factors such as diet and fluid intake, although protein intake should not be restricted, as has been conventionally endorsed for adults, as it may negatively impact children’s growth (6).

**Table 3:** Summary of treatment recommendations for pediatric stone disease based on stone size and location.

	AUA	CUA	EAU
Uncomplicated ureteral stones ≤10 mm	Observation ± medical expulsion therapy with α–-blockers	Observation ± medical expulsion therapy with α–-blockers	Observation ± medical expulsion therapy with α–-blockers
Proximal ureteral stones	SWL or URS	SWL or URS	SWL
Mid to distal ureteral stones		URS	URS
Total renal stone burden ≤20 mm	SWL or URS	SWL or URS, can consider PCNL	SWL or PCNL
Total renal stone burden >20 mm	SWL or PCNL		PCNL

SWL, shock wave lithotripsy; URS, ureteroscopy; PCNL, percutaneous nephrolithotomy; AUA, American Urological Association; CUA, Canadian Urological Association; EAU, European Association of Urology.

## 5 Future directions

Urinary drainage and decompression using a combination of nephrostomy tubes, ureteral stents, and/or urethral catheters following PCNL has been standard of care. In an effort to reduce postoperative complications and hospital length of stay, tubeless PCN—i.e., foregoing placement of a nephrostomy tube—was introduced (38). Patients with a minimal stone burden and no residual stones were candidates for this tubeless alternative and early retrospective data supported its use, finding that it was associated with decreased LOS (74). A randomized controlled trial found no differences in stone-free rates, bleeding, or postoperative complications between traditional versus tubeless PCNL in children; notably, those who underwent tubeless PCNL had a significantly shorter LOS (4.6 vs 7.7 days, p<0.001) (75). As the next iteration, the totally tubeless PCNL was introduced, which also omitted the placement of postoperative ureteral stents, thereby decreasing the likelihood of postoperative irritative voiding symptoms and eliminating the need for a repeat operation for stent removal (38). A retrospective study did not find increased incidence of postoperative complications (76). Meanwhile, a randomized controlled trial comparing traditional versus totally tubeless PCNL reported shorter LOS and decreased postoperative opioid requirements in the totally tubeless group (77). While the early success of the tubeless and totally tubeless PCNL variants is promising, studies with greater numbers of patients are warranted to define appropriate selection criteria and further characterize the safety of these approaches.

Laser technology is a mainstay of endourological techniques, particularly for stone fragmentation. Holmium lasers have been well studied for their use in ureteral and renal stone ablation, but their fiber size (minimum 200 μm) is a potential limitation as instruments are miniaturized for use in children (38, 78). Compared to holmium lasers, thulium laser fibers are emerging as the next generation tool in endourology due to its higher pulse frequency, lower pulse energy, improved energy efficiency, and smaller fiber size (50 μm), thus making it a more suitable candidate for use in conjunction with pediatric-sized instruments (e.g., micro-PCNL) and allowing for improved access to stones in difficult locations (79). A randomized controlled trial comparing use of holmium versus thulium laser fibers in adults demonstrated greater stone-free rates, shorter operative times, and decreased bleeding in the thulium laser fibers group (80). A study in the pediatric population also found higher stone-free rates and lower likelihood of retained stone fragments (OR 0.39; 95% CI 0.19–0.77) with use of thulium laser fibers (81). Current evidence seems poised to support the continued use of thulium laser fibers, however, there remains a paucity of quality studies, particularly in pediatric urology, to fully supplant the use of holmium lasers. Hence, this is a potentially exciting development in the field of endourology with implications for both adults and children.

## 6 Conclusion

Endourology has established itself as a viable, and even first-line, option for the treatment of upper urinary tract diseases in children. Numerous conditions previously managed with open procedures are now amenable to treatment with minimally invasive endourological techniques. ERBD and endopyelotomy are reasonable options for the treatment of UPJ obstruction, although bleeding from crossing vessels is a possible complication. While short-term success rates are promising and are comparable to those of pyeloplasty, the need for revision surgery is a not insignificant risk that should be properly communicated to patients and families. ERBD and endoureterotomy can be used in the management of POM, although there is emerging evidence that postoperative VUR may be a complication. Additional studies are necessary to evaluate the long-term success rates of these interventions to define the durability of these repairs. Meanwhile, SWL, URS, and PCNL have been used in the management of adult stone disease and their transition to the pediatric realm has shown similar stone-free rates. The choice of which endourologic approach to pursue is aided by guidelines but remains nuanced and is influenced by stone size and location, patient and family preferences, and physician preference and experience. Innovations in size-appropriate instrumentation and laser technology continue to make endourology a safer option for stone extraction in children.

## Author contributions

DH drafted the original manuscript. All authors contributed to the article and approved the submitted version.

## References

[1] Gobbi D Midrio P Gamba P. Instrumentation for minimally invasive surgery in pediatric urology. Transl Pediatr (2016) 5(4):186–204. doi: 10.21037/tp.2016.10.07 27867840 PMC5107385

[2] Peters CA. History of minimally invasive and robotic assisted surgery in pediatric urology. In: Gargollo PC, editor. Minimally invasive and robotic-assisted surgery in pediatric urology. Cham: Springer International Publishing (2020). p. p.3–18.

[3] Tubre RW Gatti JM. Surgical approaches to pediatric ureteropelvic junction obstruction. Curr Urol Rep (2015) 16(10):72. doi: 10.1007/s11934-015-0539-1 26275993

[4] Doudt AD Pusateri CR Christman MS. Endoscopic management of primary obstructive megaureter: A systematic review. J Endourol (2018) 32(6):482–7. doi: 10.1089/end.2017.0434 29676162

[5] Cascini V Lauriti G Di Renzo D Miscia ME Lisi G. Ureteropelvic junction obstruction in infants: Open or minimally invasive surgery? a systematic review and meta-analysis. Front Pediatr (2022) 10:1052440. doi: 10.3389/fped.2022.1052440 36507128 PMC9727311

[6] Hernandez JD Ellison JS Lendvay TS. Current trends, evaluation, and management of pediatric nephrolithiasis. JAMA Pediatr (2015) 169(10):964–70. doi: 10.1001/jamapediatrics.2015.1419 26302045

[7] Becker A Baum M. Obstructive uropathy. Early Hum Dev (2006) 82(1):15–22. doi: 10.1016/j.earlhumdev.2005.11.002 16377104

[8] Shokeir AA Nijman RJ. Antenatal hydronephrosis: Changing concepts in diagnosis and subsequent management. BJU Int (2000) 85(8):987–94. doi: 10.1046/j.1464-410x.2000.00645.x 10792193

[9] Anderson JC Hynes W. Retrocaval ureter; a case diagnosed pre-operatively and treated successfully by a plastic operation. Br J Urol (1949) 21(3):209–14. doi: 10.1111/j.1464-410x.1949.tb10773.x 18148283

[10] Liu DB Ellimoottil C Flum AS Casey JT Gong EM. Contemporary national comparison of open, laparoscopic, and robotic-assisted laparoscopic pediatric pyeloplasty. J Pediatr Urol (2014) 10(4):610–5. doi: 10.1016/j.jpurol.2014.06.010 25082711

[11] Varda BK Wang Y Chung BI Lee RS Kurtz MP Nelson CP. Has the robot caught up? national trends in utilization, perioperative outcomes, and cost for open, laparoscopic, and robotic pediatric pyeloplasty in the united states from 2003 to 2015. J Pediatr Urol (2018) 14(4):336.e1–.e8. doi: 10.1016/j.jpurol.2017.12.010 PMC6105565 29530407

[12] Bolton DM Bogaert GA Mevorach RA Kogan BA Stoller ML. Pediatric ureteropelvic junction obstruction treated with retrograde endopyelotomy. Urology (1994) 44(4):609–13. doi: 10.1016/s0090-4295(94)80073-1 7941208

[13] Sugita Y Clarnette TD Hutson JM. Retrograde balloon dilatation for primary pelvi-ureteric junction stenosis in children. Br J Urol (1996) 77(4):587–9. doi: 10.1046/j.1464-410x.1996.94520.x 8777624

[14] Tállai B Salah MA Flaskó T Tóth C Varga A. Endopyelotomy in childhood: Our experience with 37 patients. J Endourol (2004) 18(10):952–8. doi: 10.1089/end.2004.18.952 15801361

[15] Sampaio FJ. The dilemma of the crossing vessel at the ureteropelvic junction: precise anatomic study. J Endourol (1996) 10(5):411–5. doi: 10.1089/end.1996.10.411 8905485

[16] Ordóñez J Ortiz R Parente A Burgos L Fernández-Bautista B Pérez-Egido L. Long term outcome of 112 pediatric patients with ureteroplevic junction obstruction treated by endourologic retrograde balloon dilatation. Front Pediatr (2022) 10:863625. doi: 10.3389/fped.2022.863625 35547531 PMC9084922

[17] Braga LH Lorenzo AJ Skeldon S Dave S Bagli DJ Khoury AE. Failed pyeloplasty in children: Comparative analysis of retrograde endopyelotomy versus redo pyeloplasty. J Urol (2007) 178(6):2571–5; discussion 5. doi: 10.1016/j.juro.2007.08.050 17945304

[18] Kim EH Tanagho YS Traxel EJ Austin PF Figenshau RS Coplen DE. Endopyelotomy for pediatric ureteropelvic junction obstruction: A review of our 25-year experience. J Urol (2012) 188(4 Suppl):1628–33. doi: 10.1016/j.juro.2012.02.016 22906656

[19] Ceyhan E Dogan HS Tekgul S. Our experience on management of failed pediatric pyeloplasty. Pediatr Surg Int (2020) 36(8):971–6. doi: 10.1007/s00383-020-04699-9 32542506

[20] Panek W Jong T Szydełko T Chrzan R. Management of crossing vessels in children and adults: A multi-center experience with the transperitoneal laparoscopic approach. Adv Clin Exp Med (2019) 28(6):777–82. doi: 10.17219/acem/94142 30968612

[21] Lim DJ Walker RD 3rd. Management of the failed pyeloplasty. J Urol (1996) 156(2 Pt 2):738–40. doi: 10.1097/00005392-199608001-00048 8683772

[22] Corbett HJ Mullassery D. Outcomes of endopyelotomy for pelviureteric junction obstruction in the paediatric population: A systematic review. J Pediatr Urol (2015) 11(6):328–36. doi: 10.1016/j.jpurol.2015.08.014 26553288

[23] Sripathi V King PA Thomson MR Bogle MS. Primary obstructive megaureter. J Pediatr Surg (1991) 26(7):826–9. doi: 10.1016/0022-3468(91)90148-m 1895193

[24] Bujons A Saldaña L Caffaratti J Garat JM Angerri O Villavicencio H. Can endoscopic balloon dilation for primary obstructive megaureter be effective in a long-term follow-up? J Pediatr Urol (2015) 11(1):37.e1–6. doi: 10.1016/j.jpurol.2014.09.005 25748631

[25] Hendren WH. Operative repair of megaureter in children. J Urol (1969) 101(4):491–507. doi: 10.1016/s0022-5347(17)62370-x 5776032

[26] Williams DI Hulme-Moir I. Primary obstructive mega-ureter. Br J Urol (1970) 42(2):140–9. doi: 10.1111/j.1464-410x.1970.tb10013.x 5420151

[27] Angulo JM Arteaga R Rodríguez Alarcón J Calvo MJ. [Role of retrograde endoscopic dilatation with balloon and derivation using double pig-tail catheter as an initial treatment for vesico-ureteral junction stenosis in children]. Cir Pediatr (1998) 11(1):15–8. 9662865

[28] Angerri O Caffaratti J Garat JM Villavicencio H. Primary obstructive megaureter: Initial experience with endoscopic dilatation. J Endourol (2007) 21(9):999–1004. doi: 10.1089/end.2006.0122 17941775

[29] Kajbafzadeh AM Payabvash S Salmasi AH Arshadi H Hashemi SM Arabian S. Endoureterotomy for treatment of primary obstructive megaureter in children. J Endourol (2007) 21(7):743–9. doi: 10.1089/end.2006.0330 17705763

[30] Smeulders N Yankovic F Chippington S Cherian A. Primary obstructive megaureter: cutting balloon endo-ureterotomy. J Pediatr Urol (2013) 9(5):692. doi: 10.1016/j.jpurol.2013.04.010 23759477

[31] Christman MS Kasturi S Lambert SM Kovell RC Casale P. Endoscopic management and the role of double stenting for primary obstructive megaureters. J Urol (2012) 187(3):1018–22. doi: 10.1016/j.juro.2011.10.168 22264463

[32] Capozza N Torino G Nappo S Collura G Mele E. Primary obstructive megaureter in infants: Our experience with endoscopic balloon dilation and cutting balloon ureterotomy. J Endourol (2015) 29(1):1–5. doi: 10.1089/end.2013.0665 24646018

[33] García-Aparicio L Blázquez-Gómez E de Haro I Garcia-Smith N Bejarano M Martin O. Postoperative vesicoureteral reflux after high-pressure balloon dilation of the ureterovesical junction in primary obstructive megaureter. Incidence Manage Predisposing Factors World J Urol (2015) 33(12):2103–6. doi: 10.1007/s00345-015-1565-9 25899625

[34] Craig AMD Peters M Skoog SJMD Arant BSMD Jr.. Management and screening of primary vesicoureteral reflux in children (2017). Am Urological Assoc Guidelines (2017).

[35] Dwyer ME Krambeck AE Bergstralh EJ Milliner DS Lieske JC Rule AD. Temporal trends in incidence of kidney stones among children: A 25-year population based study. J Urol (2012) 188(1):247–52. doi: 10.1016/j.juro.2012.03.021 PMC3482509 22595060

[36] Routh JC Graham DA Nelson CP. Epidemiological trends in pediatric urolithiasis at united states freestanding pediatric hospitals. J Urol (2010) 184(3):1100–4. doi: 10.1016/j.juro.2010.05.018 20650479

[37] Velázquez N Zapata D Wang HH Wiener JS Lipkin ME Routh JC. Medical expulsive therapy for pediatric urolithiasis: Systematic review and meta-analysis. J Pediatr Urol (2015) 11(6):321–7. doi: 10.1016/j.jpurol.2015.04.036 PMC4688123 26165192

[38] Softness KA Kurtz MP. Pediatric stone surgery: What is hot and what is not. Curr Urol Rep (2022) 23(4):57–65. doi: 10.1007/s11934-022-01089-7 35133545

[39] Tejwani R Wang HH Wolf S Wiener JS Routh JC. Outcomes of shock wave lithotripsy and ureteroscopy for treatment of pediatric urolithiasis. J Urol (2016) 196(1):196–201. doi: 10.1016/j.juro.2016.02.2975 26997313 PMC6103480

[40] Straub M Gschwend J Zorn C. Pediatric urolithiasis: The current surgical management. Pediatr Nephrol (2010) 25(7):1239–44. doi: 10.1007/s00467-009-1394-4 PMC2874022 20130924

[41] Shokeir AA Sheir KZ El-Nahas AR El-Assmy AM Eassa W El-Kappany HA. Treatment of renal stones in children: A comparison between percutaneous nephrolithotomy and shock wave lithotripsy. J Urol (2006) 176(2):706–10. doi: 10.1016/j.juro.2006.03.080 16813924

[42] Zhang H Hong TY Li G Jiang N Hu C Cui X. Comparison of the efficacy of ultra-mini pcnl, flexible ureteroscopy, and shock wave lithotripsy on the treatment of 1-2 Cm lower pole renal calculi. Urol Int (2019) 102(2):153–9. doi: 10.1159/000493508 30352443

[43] Assimos D Krambeck A Miller NL Monga M Murad MH Nelson CP. Surgical management of stones: American urological Association/Endourological society guideline, part ii. J Urol (2016) 196):1161. doi: 10.1016/j.juro.2016.05.091 27238615

[44] Lu P Wang Z Song R Wang X Qi K Dai Q. The clinical efficacy of extracorporeal shock wave lithotripsy in pediatric urolithiasis: a systematic review and meta-analysis. Urolithiasis (2015) 43(3):199–206. doi: 10.1007/s00240-015-0757-5 25721456

[45] Caione P Collura G Innocenzi M De Dominicis M Gerocarni Nappo S Capozza N. Percutaneous endoscopic treatment for urinary stones in pediatric patients: Where we are now. Transl Pediatr (2016) 5(4):266–74. doi: 10.21037/tp.2016.09.03 PMC5107374 27867851

[46] Ozkent MS Piskin MM Balasar M Goger YE Sonmez MG. Is retrograde intrarenal surgery as safe for children as it is for adults? Urol Int (2021) 105(11-12):1039–45. doi: 10.1159/000517290 34247163

[47] Minevich E Sheldon CA. The role of ureteroscopy in pediatric urology. Curr Opin Urol (2006) 16(4):295–8. doi: 10.1097/01.mou.0000232053.74342.e9 16770131

[48] Lee JY Andonian S Bhojani N Bjazevic J Chew BH De S. Canadian Urological association guideline: Management of ureteral calculi - full-text. Can Urol Assoc J (2021) 15(12):E676–e90. doi: 10.5489/cuaj.7581 PMC8631842 34464257

[49] Önal B Kırlı EA Canpolat N Taşdemir M Gürbüz A Özman O. Different approaches among physicians to treat pediatric stone disease: A survey-based study. Arch Argent Pediatr (2021) 119(2):83–90. doi: 10.5546/aap.2021.eng.83 33749193

[50] Juliebø-Jones P Æsøy MS Gjengstø P Beisland C Ulvik Ø. Ureteroscopy for stone disease in the paediatric population: Lessons learned and outcomes in a Nordic setting. Ther Adv Urol (2022) 14:17562872221118727. doi: 10.1177/17562872221118727 36032655 PMC9403456

[51] De Dominicis M Matarazzo E Capozza N Collura G Caione P. Retrograde ureteroscopy for distal ureteric stone removal in children. BJU Int (2005) 95(7):1049–52. doi: 10.1111/j.1464-410X.2005.05464.x 15839930

[52] Anbarasan R Griffin SJ Somani BK. Outcomes and long-term follow-up with the use of ureteral access sheath for pediatric ureteroscopy and stone treatment: Results from a tertiary endourology center. J Endourol (2019) 33(2):79–83. doi: 10.1089/end.2018.0448 30511885

[53] Minevich E Defoor W Reddy P Nishinaka K Wacksman J Sheldon C. Ureteroscopy is safe and effective in prepubertal children. J Urol (2005) 174(1):276–9; discussion 9. doi: 10.1097/01.ju.0000161212.69078.e6 15947666

[54] Halinski A Steyaert H Wojciech M Sobolewski B Haliński A. Endourology methods in pediatric population for kidney stones located in lower calyx: Flexurs vs. Micro Pcnl (Microperc®) Front Pediatr (2021) 9:640995. doi: 10.3389/fped.2021.640995 34095024 PMC8175969

[55] Saad KS Youssif ME Al Islam Nafis Hamdy S Fahmy A El Din Hanno AG El-Nahas AR. Percutaneous nephrolithotomy vs retrograde intrarenal surgery for Large renal stones in pediatric patients: A randomized controlled trial. J Urol (2015) 194(6):1716–20. doi: 10.1016/j.juro.2015.06.101 26165587

[56] Baş O Dede O Aydogmus Y Utangaç M Yikilmaz TN Damar E. Comparison of retrograde intrarenal surgery and micro-percutaneous nephrolithotomy in moderately sized pediatric kidney stones. J Endourol (2016) 30(7):765–70. doi: 10.1089/end.2016.0043 26983791

[57] Mahmoud MA Shawki AS Abdallah HM Mostafa D Elawady H Samir M. Use of retrograde intrarenal surgery (Rirs) compared with mini-percutaneous nephrolithotomy (Mini-pcnl) in pediatric kidney stones. World J Urol (2022) 40(12):3083–9. doi: 10.1007/s00345-022-04186-x PMC9712365 36244014

[58] Whatley A Jones P Aboumarzouk O Somani BK. Safety and efficacy of ureteroscopy and stone fragmentation for pediatric renal stones: A systematic review. Transl Androl Urol (2019) 8(Suppl 4):S442–s7. doi: 10.21037/tau.2019.08.23 PMC6790421 31656750

[59] Satar N Zeren S Bayazit Y Aridoğan IA Soyupak B Tansuğ Z. Rigid ureteroscopy for the treatment of ureteral calculi in children. J Urol (2004) 172(1):298–300. doi: 10.1097/01.ju.0000129041.10680.56 15201799

[60] El-Assmy A Hafez AT Eraky I El-Nahas AR El-Kappany HA. Safety and outcome of rigid ureteroscopy for management of ureteral calculi in children. J Endourol (2006) 20(4):252–5. doi: 10.1089/end.2006.20.252 16646651

[61] Lesani OA Palmer JS. Retrograde proximal rigid ureteroscopy and pyeloscopy in prepubertal children: Safe and effective. J Urol (2006) 176(4 Pt 1):1570–3. doi: 10.1016/j.juro.2006.06.038 16952683

[62] Tanriverdi O Silay MS Kendirci M Kadihasanoglu M Aydin M Horasanli K. Comparison of ureteroscopic procedures with rigid and semirigid ureteroscopes in pediatric population: Does the caliber of instrument matter? Pediatr Surg Int (2010) 26(7):733–8. doi: 10.1007/s00383-010-2630-5 20521057

[63] Çitamak B Mammadov E Kahraman O Ceylan T Doğan HS Tekgül S. Semi-rigid ureteroscopy should not be the first option for proximal ureteral stones in children. J Endourol (2018) 32(11):1028–32. doi: 10.1089/end.2017.0925 30226405

[64] Grivas N Thomas K Drake T Donaldson J Neisius A Petřík A. Imaging modalities and treatment of paediatric upper tract urolithiasis: A systematic review and update on behalf of the eau urolithiasis guidelines panel. J Pediatr Urol (2020) 16(5):612–24. doi: 10.1016/j.jpurol.2020.07.003 32739360

[65] Tekgül S Stein R Bogaert G Nijman RJM Quaedackers J t Hoen L. European Association of urology and European society for paediatric urology guidelines on paediatric urinary stone disease. Eur Urol Focus (2022) 8(3):833–9. doi: 10.1016/j.euf.2021.05.006 34052169

[66] Ozden E Mercimek MN. Percutaneous nephrolithotomy in pediatric age group: assessment of effectiveness and complications. World J Nephrol (2016) 5(1):84–9. doi: 10.5527/wjn.v5.i1.84 PMC4707172 26788467

[67] Senocak C Ozbek R Bozkurt OF Unsal A. Predictive factors of bleeding among pediatric patients undergoing percutaneous nephrolithotomy. Urolithiasis (2018) 46(4):383–9. doi: 10.1007/s00240-017-1001-2 28702679

[68] Çıtamak B Altan M Bozacı AC Koni A Doğan HS Bilen CY. Percutaneous nephrolithotomy in children: 17 years of experience. J Urol (2016) 195(4 Pt 1):1082–7. doi: 10.1016/j.juro.2015.11.070 26682755

[69] Ozden E Sahin A Tan B Doğan HS Eren MT Tekgül S. Percutaneous renal surgery in children with complex stones. J Pediatr Urol (2008) 4(4):295–8. doi: 10.1016/j.jpurol.2008.01.212 18644533

[70] Jones P Bennett G Aboumarzouk OM Griffin S Somani BK. Role of minimally invasive percutaneous nephrolithotomy techniques-micro and ultra-mini pcnl (<15f) in the pediatric population: A systematic review. J Endourol (2017) 31(9):816–24. doi: 10.1089/end.2017.0136 28478724

[71] Davis RB Farber NJ Kaplan A Patel R Steckler RE Elsamra SE. Contemporary practice patterns in the treatment of pediatric stone disease. Can J Urol (2018) 25(4):9427–32. 30125525

[72] Farouk A Tawfick A Shoeb M Mahmoud MA Mostafa DE Hasan M. Is mini-percutaneous nephrolithotomy a safe alternative to extracorporeal shockwave lithotripsy in pediatric age group in borderline stones? a randomized prospective study. World J Urol (2018) 36(7):1139–47. doi: 10.1007/s00345-018-2231-9 29450731

[73] Grabsky A Tsaturyan A Musheghyan L Minasyan G Khachatryan Y Shadyan G. Effectiveness of ultrasound-guided shockwave lithotripsy and predictors of its success rate in pediatric population: A report from a national reference center. J Pediatr Urol (2021) 17(1):78.e1–.e7. doi: 10.1016/j.jpurol.2020.10.014 33153916

[74] Akman T Binbay M Yuruk E Sari E Seyrek M Kaba M. Tubeless procedure is most important factor in reducing length of hospitalization after percutaneous nephrolithotomy: Results of univariable and multivariable models. Urology (2011) 77(2):299–304. doi: 10.1016/j.urology.2010.06.060 20970842

[75] Song G Guo X Niu G Wang Y. Advantages of tubeless mini-percutaneous nephrolithotomy in the treatment of preschool children under 3 years old. J Pediatr Surg (2015) 50(4):655–8. doi: 10.1016/j.jpedsurg.2014.11.042 25840082

[76] Ozturk A Guven S Kilinc M Topbaş E Piskin M Arslan M. Totally tubeless percutaneous nephrolithotomy: Is it safe and effective in preschool children? J Endourol (2010) 24(12):1935–9. doi: 10.1089/end.2010.0100 20815757

[77] Aghamir SM Salavati A Aloosh M Farahmand H Meysamie A Pourmand G. Feasibility of totally tubeless percutaneous nephrolithotomy under the age of 14 years: A randomized clinical trial. J Endourol (2012) 26(6):621–4. doi: 10.1089/end.2011.0547 22192104

[78] Aldoukhi AH Roberts WW Hall TL Ghani KR. Holmium laser lithotripsy in the new stone age: Dust or bust? Front Surg (2017) 4:57. doi: 10.3389/fsurg.2017.00057 29067287 PMC5649137

[79] Gao B Bobrowski A Lee J. A scoping review of the clinical efficacy and safety of the novel thulium fiber laser: The rising star of laser lithotripsy. Can Urol Assoc J (2021) 15(2):56–66. doi: 10.5489/cuaj.6804 32744995 PMC7864720

[80] Ulvik Ø Æsøy MS Juliebø-Jones P Gjengstø P Beisland C. Thulium fibre laser versus Holmium:Yag for ureteroscopic lithotripsy: Outcomes from a prospective randomised clinical trial. Eur Urol (2022) 82(1):73–9. doi: 10.1016/j.eururo.2022.02.027 35300888

[81] Jaeger CD Nelson CP Cilento BG Logvinenko T Kurtz MP. Comparing pediatric ureteroscopy outcomes with superpulsed thulium fiber laser and low-power Holmium:Yag laser. J Urol (2022) 208(2):426–33. doi: 10.1097/ju.0000000000002666 35703000

